# Dancing attraction: followers of honey bee tremble and waggle dances exhibit similar behaviors

**DOI:** 10.1242/bio.025445

**Published:** 2017-05-11

**Authors:** Calvin Lam, Yanlei Li, Tim Landgraf, James Nieh

**Affiliations:** 1 University of California San Diego, Section of Ecology, Behavior, and Evolution, 9500 Gilman Drive, MC0116, La Jolla, CA 92093-0116, USA; 2 Freie Universität Berlin, Fachbereich Mathematik und Informatik, Institut für Informatik, Arnimallee 7, Berlin 14195, Germany

**Keywords:** *Apis mellifera*, Foraging communication, Signaling, Colony organization, Division of labor

## Abstract

The function of the honey bee tremble dance and how it attracts signal receivers is poorly understood. We tested the hypothesis that tremble followers and waggle followers exhibit the same dance-following behavior. If correct, this could unify our understanding of dance following, provide insight into dance information transfer, and offer a way to identify the signal receivers of tremble dance information. Followers showed similar initial attraction to and tracking of dancers. However, waggle dancers were faster than tremble dancers, and follower-forward, -sideways, and -angular velocities were generally similar to the velocities of their respective dancers. Waggle dancers attracted followers from 1.3-fold greater distances away than tremble dancers. Both follower types were attracted to the lateral sides of dancers, but tremble followers were more attracted to the dancer's head, and waggle followers were more attracted to the dancer's abdomen. Tremble dancers engaged in 4-fold more brief food exchanges with their followers than waggle dancers. The behaviors of both follower types are therefore relatively conserved. Researchers can now take the next steps, observing tremble followers to determine their subsequent behaviors and testing the broader question of whether follower attraction and tracking is conserved in a wide range of social insects.

## Abstract (precis)

**Summary:** We show that tremble dance and waggle dance followers exhibit similar orientation and tracking behaviors, suggesting that signal detection via following is a conserved honey bee behavior.

## INTRODUCTION

In social insects, communication plays a key role in coordinating colony life and fitness (Dornhaus et al., 2006; Hölldobler and Wilson, 1990; Hunt and Richard, 2013; Sherman and Visscher, 2002). In multiple cases, information is transferred by a signaler and a follower tracking each other. Ant tandem running allows the follower to find a food source by following the leader (Franks and Richardson, 2006). In termites, tandem running allows males to pursue females to a burrow (Nalepa and Jones, 1991). In both of these cases, the receiver physically follows a sender to a location. The famous honey bee waggle dance provides a referential example in which the follower decodes the information provided in the waggle dance to learn the direction, distance, and relative quality of a resource (Schürch and Ratnieks, 2016; von Frisch, 1967). A waggle dancer performs figure-eight-like motions centered on a waggle run that encodes distance and direction (Božič and Valentinčič, 1991; von Frisch, 1967). Although there is inter-dance variation in the waggle dance (Schürch et al., 2016), the dance itself is highly recognizable due to its stereotyped movements (Landgraf et al., 2011; Rohrseitz and Tautz, 1999; von Frisch, 1967).

There is another honey bee dance that is often seen inside the hive and has been described for nearly a century (von Frisch, 1923), but whose functions remain somewhat unclear (Schneider and Lewis, 2004) and whose information receivers have yet to be fully identified: the tremble dance (Zittertanz). In general, the tremble dance appears in a wide variety of contexts that are associated with deteriorating foraging conditions (Kirchner and Lindauer, 1994). A tremble dancer traverses the comb with highly irregular running, shaking, vibrating, and trembling motions while rotating about its body axis (Nieh, 1993; Seeley, 1992; von Frisch, 1923). Unlike the waggle dance, tremble dance movements are far less stereotyped (Seeley, 1992; von Frisch, 1967). The function of the tremble dance was unknown (Lindauer, 1948). In fact, von Frisch (1967) wrote ‘it may be deduced that the trembling dance gives the hivemates no information and they pay no attention to it. It occurs as the result of adverse circumstances and experiences and perhaps is comparable to the condition that Florey (1954) has described as neurosis, which is seen when a situation of nervous conflict is produced artificially in bees’.

However, Seeley (1992) discovered that the tremble dance can act as a signal and draw the attention of followers. He showed that foragers would tremble dance if they experienced a long food-unloading delay inside the nest. Returning nectar foragers usually transfer their collected nectar to food processing bees, which we will call ‘unloaders’ (not ‘nectar receivers’, to avoid confusion with the term ‘signal receivers’). If there is a sudden influx of nectar, the unloading wait time can increase due to a lack of available unloaders, and the colony should rebalance its division of labor. Bees have evolved an elegant solution via the tremble dance. The probability of a forager tremble dancing, instead of waggle dancing, increases the longer the forager must wait to be unloaded. The conversion from waggle dancing to tremble dancing therefore helps to reduce nectar influx and can also function to increase the number of food unloaders (Seeley, 1992). Tremble dancers may recruit bees to become food unloaders, though it is unclear exactly how the behavior of tremble followers changes immediately after they follow a tremble dancer (Seeley, 1992). Once balance is restored, forager unloading wait times decrease and waggle dancing can resume (Seeley, 1992).

The same honey bee signal can be used in multiple contexts (von Frisch, 1967), and tremble dancing does not stem solely from food unloading delays (Biesmeijer, 2003; Couvillon, 2012). As von Frisch (1967) pointed out, tremble dancing can be triggered when a bee has an aversive experience at a food source. Foragers that consumed poisoned sucrose solution (Lindauer, 1948; Schick, 1953; Schneider, 1949) or very salty sucrose solution (Kirchner and Lindauer, 1994) were more likely to tremble dance. Crowding at a feeder (Kirchner, 1993), in the absence of unloading delays, increased the probability of the forager tremble dancing (Lau and Nieh, 2010; Thom, 2003). Bees attacked at a food source were more likely to tremble dance and produced stop signals, which inhibit waggle dancing for dangerous food (Nieh, 2010). In our experiments, we focused on a specific, highly replicable context, tremble dancing elicited by attacks at a food source. This allowed us to compare, by alternating between focal foragers for the same food source, tremble dancers to waggle dancers.

To understand how the tremble dance helps to regulate colony foraging, we need to identify the signal receivers, the dance followers, beginning with their initial attraction to the dancer. A follower is a bee that, after turning towards and approaching the dancer, tracks the dancer as it moves by keeping its head facing and adjacent to the waggle dancer's body (Al Toufailia et al., 2013; Božič and Valentinčič, 1991; Landgraf et al., 2011; Nieh, 1993; Rohrseitz and Tautz, 1999; Tautz and Rohrseitz, 1998; von Frisch, 1967) or the tremble dancer's body (Seeley, 1992). The term ‘follower’ has primarily been applied to bees that track waggle dancers (von Frisch, 1967), but has also been used for bees that orient towards tremble dancers (Seeley, 1992). Demonstrating that tremble followers behave similarly to waggle followers can help identify the receivers of tremble dance information because waggle following is essential for signal receipt (von Frisch, 1967).

The behavior of waggle followers is quite conspicuous. Waggle followers track waggle dancers quite closely and are clustered around the dancer's abdomen (Tautz and Rohrseitz, 1998). Waggle followers often make contact with the body of the waggle dancer (Božič and Valentinčič, 1991), and followers can become strongly attracted to dancers after initial contact between follower antennae and dancer body (Tautz and Rohrseitz, 1998). However, waggle followers can also be attracted from a distance, potentially by the attractive odors produced by waggle dancers (Thom et al., 2007), the near-field sounds (Michelsen, 2014), or weak substrate vibrations generated by a waggle dancer (Nieh and Tautz, 2000).

In contrast, little information exists about tremble followers. Seeley (1992) provides a short description: ‘a tremble dancing bee clearly attracts the attention of bees immediately adjacent to it. These nearby bees frequently will turn to face the dancer and will touch it with their antennae. They may maintain contact with the dancer for a few seconds (rarely more than 5 s), walking along behind it for several centimeters (rarely more than 5 cm)’. Subsequently, these followers typically moved away from the dancer, but there appeared to be no noticeable change in their activity level shortly after contact with a tremble dancer.

This description of tremble following is interesting because it is quite similar to our understanding of waggle following. We therefore hypothesized that tremble following and waggle following are essentially the same behavior. Testing this hypothesis provides insight into how dance information is transmitted in honey bees and, more broadly, yields insight into following in social insects, a behavior that is used to transfer multiple kinds of information (Franks and Richardson, 2006; Nalepa and Jones, 1991). In addition, the ability to reliably identify tremble followers is an important step in understanding the function of the tremble dance in its different contexts.

Testing this hypothesis requires a detailed quantitative analysis, similar to those conducted with waggle following (Al Toufailia et al., 2013; Božič and Valentinčič, 1991; Landgraf et al., 2011; Nieh, 1993; Rohrseitz and Tautz, 1999; Tautz and Rohrseitz, 1998; von Frisch, 1967). Furthermore, it would be good to apply the same analysis criteria to both behaviors as performed by foragers from the same colony that are studied under the same conditions. We therefore measured follower behavior in great detail and compared the behaviors of followers orienting to tremble dancers and waggle dancers. We used multiple colonies and, to facilitate comparisons, had bees forage at a standardized stimulus, a rich 2.5 M sucrose solution located 100 m from each colony.

## RESULTS

### Both dance types were recorded for similar durations under similar conditions of bee density and video frame size

There was no significant difference in duration tracked between waggle dances and tremble dances for our video analyses (*F*_1,42_=0.52, *P*=0.47, <1% colony effect). There was also no significant difference between bee densities during waggle dances or tremble dances (*F*_1,48_=1.32, *P*=0.26, 3% colony effect) or between the dance floor area filmed (number of horizontal cells, fixed aspect ratio) during videos of waggle dancing or tremble dancing (*F*_1,83_=1.69, *P*=0.20, 30% colony effect). On average, there were 2.9±2.6 waggle followers per waggle performance and 2.6±1.9 tremble followers per tremble performance. Tremble dancers engaged in significantly higher levels of trophallaxis with their followers than waggle dancers with their followers (0.12±0.25 trophallaxes follower^−1^ tremble dance^−1^ and 0.03±0.14 trophallaxes follower^−1^ waggle dance^−1^: *F*_1,49_=4.78, *P*=0.03, <1% colony effect).

### Waggle dancers had the highest rates of absolute motion

**Fig. 1.**
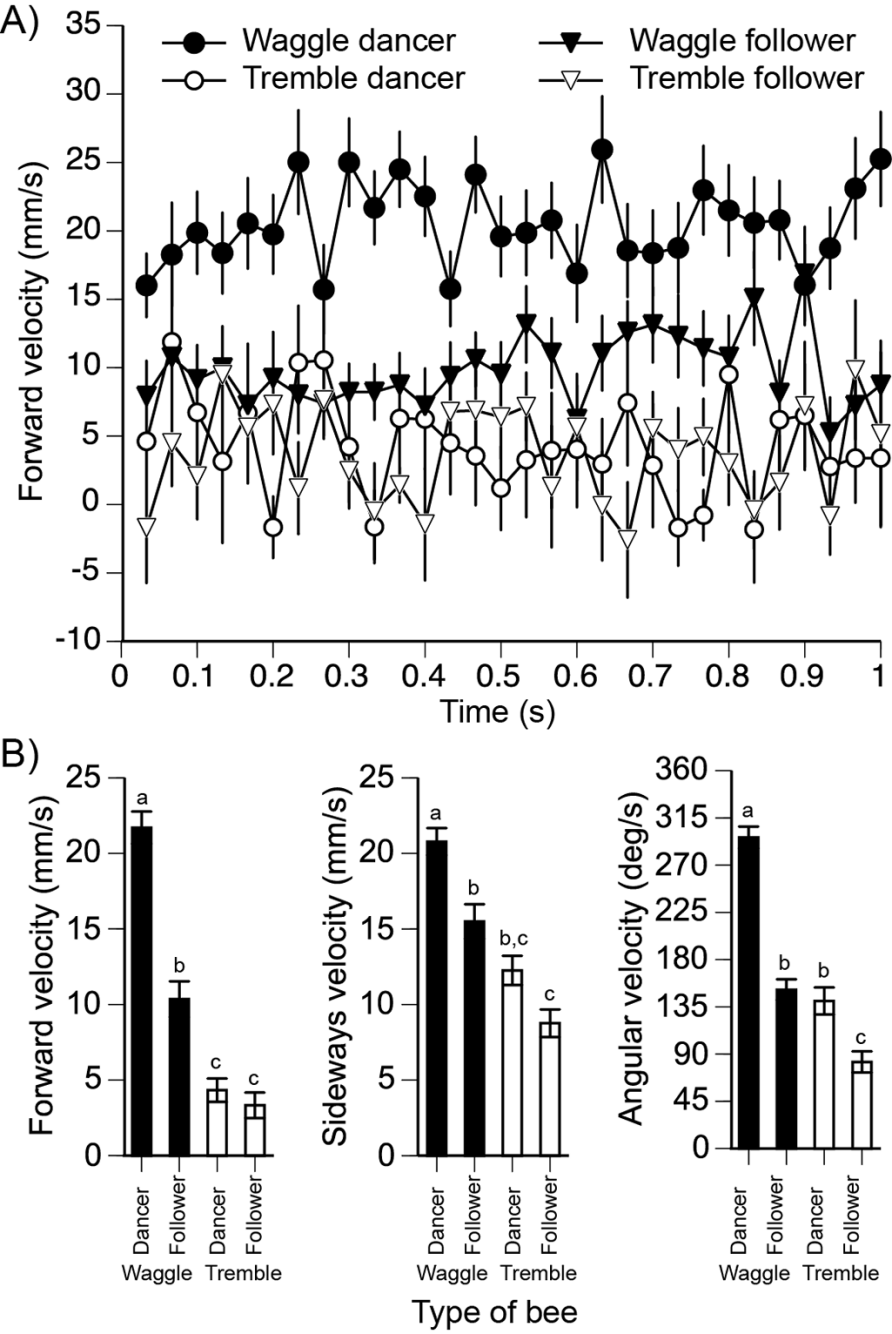
**Absolute bee velocities.** (A) Forward (longitudinal) velocity of different bee types over time. Positive values indicate forward motion. Negative values indicate backward motion. This plot demonstrates that most motion was forward, as expected. (B) Because there was no effect of time, we present the mean values of forward velocity, sideways velocity, and angular velocity for each bee type. Plots show mean±s.e.m. and differences are indicated with different letters as calculated from the Repeated-Measures model (Tukey's HSD test, *P*<0.05): *N*_colonies_=4; *N*_tremble dances_=18; *N*_tremble followers_=31; *N*_waggle dances_=44; *N*_waggle followers_=96.

We examined the motions of four different types of bees: waggle dancers, waggle followers, tremble dancers, and tremble followers. There was a significant effect of bee type (*F*_3,202_=55.59, *P*<0.0001), but no significant effect of time (*F*_1,4479_=0.05, *P*=0.82), and no significant interaction of bee type×time (*F*_3,4584_=1.24, *P*=0.29) on forward velocity, the movement along the longitudinal axis of the bee (Fig. 1A). Colony accounted for 2% of model variance. Waggle dancers had significantly higher forward velocities than any other group, and tremble dancers and followers did not have significantly different forward velocities (Tukey's HSD test, *P*<0.05, Fig. 1B).

Similarly, for sideways velocity, there was a significant effect of bee type (*F*_3,183_=21.59, *P*<0.0001), but no significant effect of time (*F*_1,4775_=0.26, *P*=0.61), and no significant interaction of bee type×time (*F*_3,4842_=0.83, *P*=0.48). Colony accounted for 0.2% of model variance. Waggle dancers had significantly higher sideways velocities than any other group, and tremble dancers and followers did not have significantly different sideways velocities (Tukey's HSD test, *P*<0.05, Fig. 1B).

Finally, for angular velocity, there was a significant effect of bee type (*F*_3,195_=95.65, *P*<0.0001), but no significant effect of time (*F*_1,4377_=0.01, *P*=0.91), and no significant interaction of bee type×time (*F*_3,4495_=0.32 *P*=0.82). Colony accounted for <0.1% of model variance. Waggle dancers had the highest angular velocities, followed by waggle followers and tremble dancers (not significantly different) and tremble followers (Tukey's HSD test, *P*<0.05, Fig. 1B).

### Waggle dancers drew in followers from greater distances than tremble dancers

**Fig. 2.**
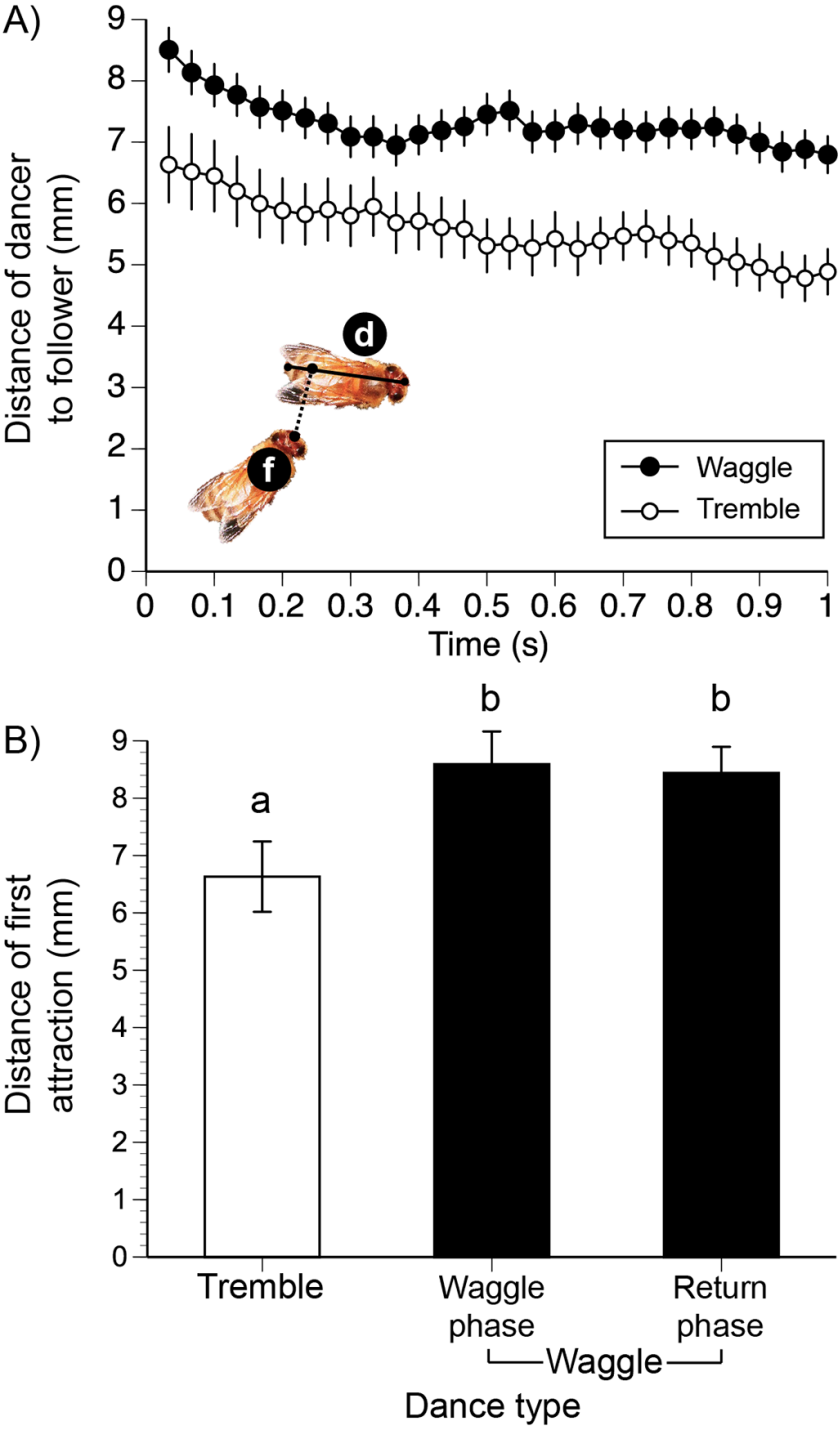
**Distance between followers and dancers.** (A) Distances between the head of the follower (f) and the closest point on the body of the dancer (d) over time. For both dance types, there was a significant effect of time (*P*<0.0001^DS^) such that tremble followers and dance followers drew closer to the dancer that they were following. However the slopes of the change of distance with time were different, depending upon the dance type (interaction time×dance type, *P*=0.005^DS^). (B) The distance of first attraction is the distance between follower and dancer in which a follower was first attracted to a dancer. There was no significant difference between the distance of first attraction to the waggle phase or the return phase of waggle dancers. Plots show mean±s.e.m. In the bar graphs, significant differences are indicated with different letters (Tukey's HSD test, *P*<0.05): *N*_colonies_=4; *N*_tremble dances_=25; *N*_tremble followers_=64; *N*_waggle dances_=61; and *N*_waggle followers_=174.

For both dance types, followers drew closer to the dancers over time (Fig. 2A). There was no significant difference between the attraction distances of followers attracted to waggle dancers during the waggle or return phases (Tukey's HSD test, *P*>0.05, Fig. 2B). We therefore compared following of waggle dances (both phases pooled) with following of tremble dances. Tremble dancers attracted followers from significantly shorter distances than waggle dancers (*k*=2, first attraction distance: *F*_1,164_=8.34, *P*=0.004^DS^, 8% colony effect; distance over all frames: *F*_1,171_=14.42, *P*=0.0002^DS^, 4% colony effect, Fig. 2B). There was a significant effect of time (*F*_1,6900_=66.20, *P*<0.0001^DS^) and the interaction time×dance type (*F*_1,6900_=7.87, *P*=0.005^DS^) because the slopes of follower-dancer distances were significantly different for waggle dances as compared to tremble dances (−0.94 mm s^−1^ for waggle and −1.55 mms^−1^ for tremble dances).

### Tremble and waggle follower are positioned differently around dancers

**Fig. 3.**
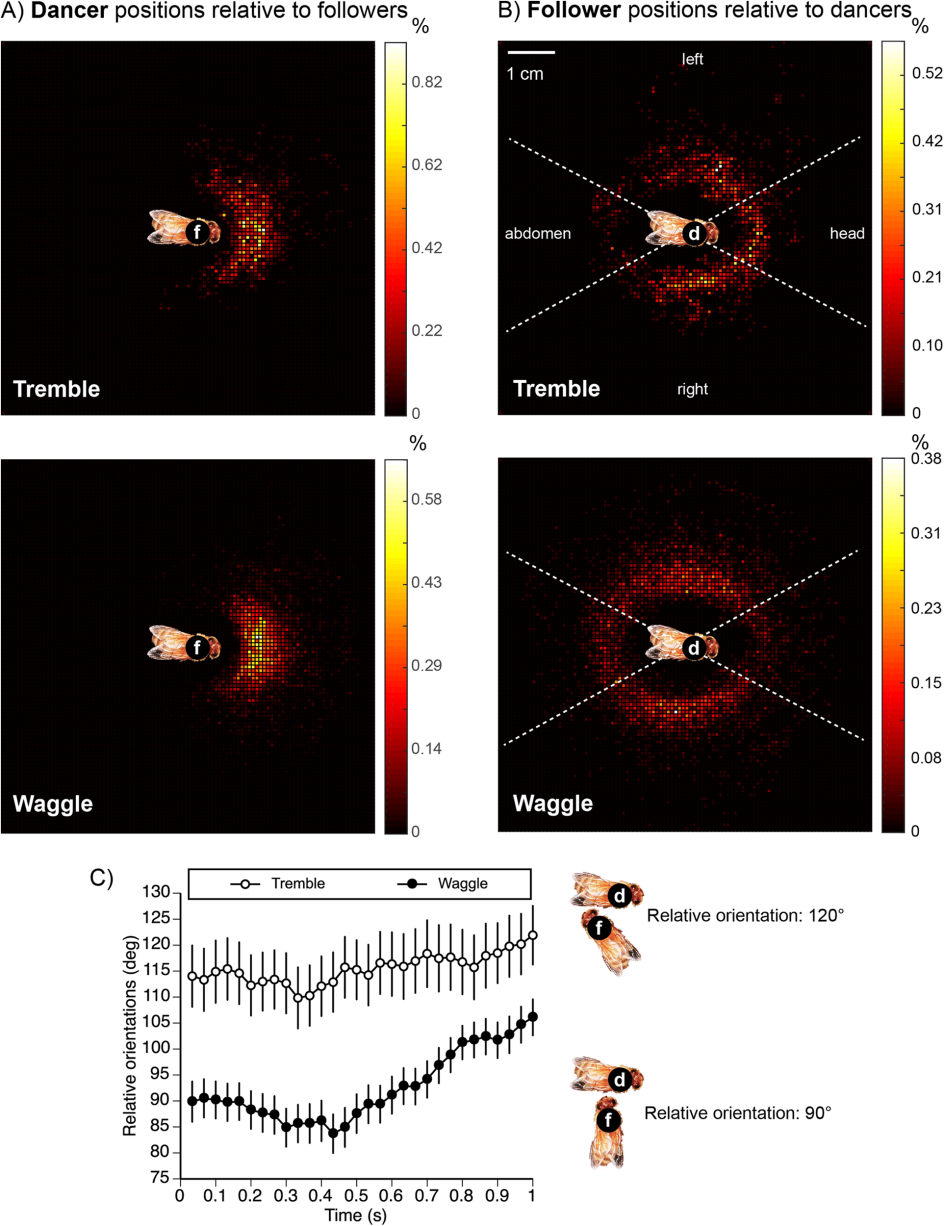
**Followers are distributed differently around tremble dancers and waggle dancers, and their relative orientations change over time.** Data are from all dancers (d) and all followers (f). The inset bee images are slightly reduced in scale to avoid obscuring data points, but show the correct centering and orientation. (A) If we fix follower's position in each video frame, we see that tremble and waggle followers both tend to face their respective dancers. (B) By fixing the dancer's position, we see that tremble followers tend to be positioned around the dancer's head and sides while waggle followers tend to be positioned around the dancer's abdomen and sides. The dashed white lines show the quadrants used in our analyses. (C) The orientation angle of the followers relative to the dancers. Plots show mean±s.e.m. orientations: *N*_colonies_=4; *N*_tremble dances_=25; *N*_tremble followers_=64; *N*_waggle dances_=61; *N*_waggle followers_=174.

Followers of waggle and tremble dances generally positioned themselves facing their dancer (Fig. 3A). However, waggle and tremble followers positioned themselves differently around their dancers. Tremble followers tended to position themselves around the dancer's head and sides. Waggle followers tended to position themselves around the dancer's abdomen and sides (Fig. 3B).

These differences were confirmed by our quadrant analyses. We first examined initial attraction. There was no difference between the initial attraction of followers to the lateral sides (left or right) of tremble or waggle dancers (*k*=3, L-R *χ*^2^=0.15, 1 d.f., *P*=0.69). However, waggle followers were more likely to be initially attracted by the dancer's abdomen, whereas tremble followers were more likely to be initially attracted by the dancer's head (L-R *χ*^2^=7.17, 1 d.f., *P*=0.007^DS^, Fig. 3A). Colony was not a significant factor (L-R *χ*^2^≤0.84, 3 d.f., *P*≥0.84).

In the repeated-measures model that compared head versus abdomen quadrant orientation over time, there were significant effects of dancer type (*k*=3, L-R *χ*^2^=80.62, 1 d.f., *P*<0.0001^DS^), time (L-R *χ*^2^=9.54, 1 d.f., *P*=0.002^DS^), and the interaction dancer type×time (L-R *χ*^2^=10.68, 1 d.f., *P*=0.001^DS^). This occurred because waggle and tremble followers shifted their quadrant positions differently over time (different slopes, see analysis below, Fig. 3C). Colony was not a significant factor (L-R *χ*^2^≤6.64, 3 d.f., *P*≥0.08).

We then calculated how the relative orientations of dancers and followers changed over time (Fig. 3C). The relative orientations changed significantly over time (*k*=3, dancer type effect: *F*_1,68_=19.88, *P*<0.0001^DS^; time effect: *F*_1, 6900_=59.92, *P*<0.0001^DS^). There was a significant effect of the interaction dancer type×time (*F*_1,6900_=14.03, *P*=0.002^DS^) because the relative orientations differed in slope over time. Colony accounted for 0.1% of model variance. Tremble followers oriented towards tremble dancers at a fairly constant angle of 115-120° over time. Waggle followers initially adopted a roughly perpendicular orientation (90°) but then shifted to about 105° over time (Fig. 3C). The strong separation between the relative orientations of tremble followers to tremble dancers as compared to waggle followers to waggle dancers is notable.

## DISCUSSION

Our results support the hypothesis that tremble and waggle following are similar. Overall, tremble followers behaved similarly to waggle dancers. They matched the movement pace of the dances that they followed (Fig. 1B) and the majority oriented to the sides of the dancer (Fig. 3B). However, there were some differences. Waggle dancers drew in followers from a 1.3-fold greater mean distance (8.5 mm) than tremble dancers (6.6 mm). Waggle dancers moved significantly more rapidly in all velocity measures (forward, sideways, and angular velocities) than tremble dancers. Likewise, waggle dance followers also moved more rapidly in all of these measures than tremble dance followers, as expected given that waggle dance followers were tracking faster movements.

Tremble dance followers faced the head and sides of tremble dancers (as shown in Seeley, 1992), but waggle dance followers faced the rear of the waggle dancer's abdomen and its sides (see below). Thus, the sides of the dancer were approximately equally attractive to both tremble and waggle followers. However, tremble followers were more attracted to the head of the tremble dancer, and waggle followers were more attracted to the tip of the waggle dancer's abdomen. The higher forward velocity of waggle dancers (Fig. 1B) as compared to tremble dancers could have contributed to fewer followers being positioned around the head of the waggle dancer (Fig. 3B) if followers were more easily pushed away from the dancers head during dancing. However, greater trophallaxis with tremble dancers as compared to waggle dancers may also have played a role.

Like Božič and Valentinčič (1991), we found that waggle followers initially approached and tracked dancers from the left and right sides of the dancer (Fig. 3B). Božič and Valentinčič (1991) further reported that waggle followers tended to remain perpendicular to the dancer, which we also observed within the first 0.5 s of waggle dance following (Fig. 3C). Our data on waggle follower positions is therefore similar to those reported by other researchers (Judd, 1995; Łopuch and Tofilski, 2017; Tautz and Rohrseitz, 1998). Judd (1995) suggested that a position behind the waggle dancer is important for followers to successfully receive dance information. However, followers positioned lateral or posterior to the dancer abdomen are also able to obtain dance information (Tanner and Visscher, 2009).

Although both waggle and tremble dancers had approximately the same number (2.6-2.8) of followers per dance, tremble dancers performed trophallaxis with followers at a 4-fold higher rate per follower than waggle dancers. This could also explain why the head of the tremble dancer was more attractive to tremble followers than the head of the waggle dancer to waggle followers. It is unclear why trophallaxis was more commonly performed by tremble dancers, but we observed that tremble dancers could engage in brief food exchanges while dancing. Reliably determining the direction of food exchange is unfortunately not possible with our data because it requires detailed imaging, very close up or higher-resolution videos, of the heads of the food exchangers. Close-up videos would have reduced our ability to record follower behaviors. However, we suspect that tremble dancers were largely food donors.

In our experiment, we allowed foragers to feed before attacking them. When the attacked bees returned to the nest, instead of seeking food unloaders, most immediately began to tremble dance. Many tremble dancers therefore had a store of unloaded food. If tremble dancing conveys a warning, then the odors naturally associated with the dangerous food could be conveyed, in addition to diffusion from abdominal hairs, through such trophallactic events. Alternatively, in the context of an excessive nectar influx and insufficient food unloaders (Seeley, 1992), such trophallaxis may reinforce the message that more food unloaders are required. These results suggest the intriguing possibility that trophallaxis is part of how tremble dance information is imparted to receivers. However, this hypothesis requires further testing.

It is unclear why waggle dancers attracted followers from greater distances than tremble dancers. Initial observations suggested that waggle dancers moved more vigorously, and this was borne out by our analyses, which showed higher waggle dancer velocities in all measures (Fig. 1). Tactile contact, such as antennal contact, likely plays a role in tremble attraction as it does in waggle attraction (Tautz and Rohrseitz, 1998). However, the majority of tremble followers were attracted before any physical contact with the dancer.

How were tremble followers attracted? There are several possible explanations. Waggle dancers produce air vibrations (Michelsen et al., 1987) and weak comb vibrations (Nieh and Tautz, 2000). Tremble dancers may also vibrate the comb, though this remains to be determined. For all of our video recordings, we tracked waggle and tremble dancers with a microphone held approximately 1 cm above the center of the dancer's body. Waggle dancers typically produced detectable near-field sounds, but tremble dancers did not. However, attraction to near-field dance sounds probably does not explain the attraction differences between tremble and waggle dances because we found no difference between the distance of first attraction to the waggle phase or the silent return phase of waggle dances (Fig. 2B). Similarly, Tautz and Rohrseitz (1998) showed that the sound-producing waggle phase did not attract followers from greater distances away then the silent return phase. Olfactory attraction provides another explanation, given that waggle dancers emit attractive odors from their abdomens (Thom et al., 2007). Tremble dancers may also produce such attractive odors, though this remains to be determined. Finally, waggle dancers can have elevated body temperatures (Stabentheiner and Hagmüller, 1991), which may also be attractive, particularly if elevated temperatures increase the amount of volatilized waggle odors. In general, the more vigorous motions displayed by waggle dancers as compared to tremble dancers, may also facilitate attraction by increasing body temperature, scent volatilization and comb vibration amplitudes, though demonstrating this requires further study.

Tautz and Rohrseitz (1998) studied the first attraction distances of waggle followers and used a slightly different methodology, measuring the distance between the head of the follower and the closest body point (not including appendages) of the dancer. On open cells (as in our study), they found mean attraction distances of 10-17 mm. In contrast, we found a mean attraction distance of 8.5 mm for waggle followers. However, our combs had a significantly higher density of bees (1.2-fold higher, Wilcoxon signed rank test, *W*=955.5, *P*<0.0001). Our shorter attraction distances may therefore have resulted from a more crowded comb. It is unclear how to correct for such a higher density, but applying a 1.2-fold linear correction factor to our mean waggle follower attraction distances yields 10.2 mm, which is closer to the range observed by Tautz and Rohrseitz (1998).

Another possible explanation is that we used a 7.5% smaller field of view than that of Tautz and Rohrseitz (1998) and could therefore have missed bees that were attracted from greater distances. However, 7.5% is relatively small difference. Even with these methodological differences, our overall results on waggle following are quite similar. Like Tautz and Rohrseitz (1998), we found no significant difference between the distances of first attraction to either the waggle or return phases of the waggle dance (Fig. 2B) and the majority of followers were laterally attracted to the left and right quadrants of waggle dancers (Fig. 3B). Because we applied our methods uniformly to waggle and tremble dances performed by foragers at approximately the same time of day from the same colonies for identical food sources, and with the same sucrose concentrations at the same distance, the differences that we found between waggle and tremble following are likely robust.

At a colony level, the greater attraction distance of followers to waggle as compared to tremble dancers may be mitigated by the much larger area that tremble dancers cover. Each waggle dance is localized to an area of approximately 28 mm^2^ within the dance floor of the nest (Landgraf et al., 2011) and seldom persists for more than a few minutes. In contrast, a tremble dance can span 100 cm^2^ within 2 min, ranges widely throughout the brood areas of the nest and the dance floor, and lasts an average of 27 min (Seeley, 1992). Tremble dancers can therefore reach a wider audience and travel throughout the nest. Seeley (1992) did not observe a noticeable change in tremble follower activity within 1-2 min of contact with a tremble dancer. However, it should now be possible to identify and track tremble followers who exhibit stronger following behavior (attraction from a greater distance, trophallaxis, tighter tracking, and closer dancer-follower velocity matching) over longer periods to solve the mystery of precisely how tremble dance receipt affects follower behavior.

In many social insects, including ants (Franks and Richardson, 2006), termites (Nalepa and Jones, 1991), and stingless bees (Hrncir and Barth, 2014; Nieh, 2009), receivers are attracted to and follow signalers to gain important information. A broader study of following in social insects – the cues and signals that elicit initial attraction, the sensory modalities involved, and the mechanisms that allow maintenance of proximity – could therefore be informative. Within the same species, an intriguing question is whether the neural mechanisms that facilitate follower orientation to one signal provide a pathway for the evolution of following and attending to new signals. This could be a form of sensory exploitation, not in the context of sexual selection, but rather in context of cooperative signaling within a collective.

## MATERIALS AND METHODS

### Study site and observation colonies

We conducted our study at the UC San Diego Biological Field Station in La Jolla, California, USA, between July-October 2007 and July-September 2011. We used four healthy colonies of *Apis mellifera ligustica* (two colonies in 2007 and two in 2011), each containing approximately 4000 bees (determined by photographic estimation). Each colony was housed in a temperature-controlled room (30°C) within a three-frame observation hive with one entrance tunnel leading outside. We recorded both waggle dancing and tremble dancing on each trial day between 09:00 h and 16:00 h.

### Training

We trained approximately five bees at a time to a grooved plate feeder located 100 m away from the hive (method of von Frisch, 1967) that provided unscented 2.5 M sucrose solution (66% w v^−1^) *ad libitum*. Bees were individually marked at the feeder with a unique combination of acrylic paint colors on the thorax, abdomen, or both (method of von Frisch, 1967). We used aspirators to remove excess bees and thereby maintained a fairly constant nectar flow from the feeder. Foraging for natural food sources also occurred, but was limited. We chose seasons of relative food dearth, because it is difficult to train bees to artificial feeders when there are abundant natural food sources. Thus, nectar inflow into each colony came largely from the feeder.

We randomly selected some bees and induced tremble dancing (Nieh, 2010) by pinching a forager's right metathoracic leg at the basitarsus for 1 s, imitating the attacks of *Vespula pensylvanica* wasps on foraging honey bees (Jack-McCollough and Nieh, 2015). Bees were allowed to feed before pinching so that they could engage in trophallaxis upon return to the hive. Tremble dancing was therefore induced by an aversive stimulus, not by a change in food influx, because a fixed number of bees visited the feeder and access to natural food sources did not dramatically shift during a given trial day. Bees were not harmed by this pinching and could return to the nest where they walked and performed complex motor activities such as waggle dancing and tremble dancing. To clean all apparatus and remove potential odors, we used laboratory detergent followed by a 100% wash of ethanol and several hours of drying at the end of each trial.

### Video recordings

Foragers were filmed inside the hive at 30 frames per second (fps) with a Sony HDR-HC7 high definition camcorder. To better analyze the behaviors of dancers and followers, we focused on extended performances: waggle dances that consisted of more than 14 waggle runs and tremble dances that were >40 s in duration. In total, we analyzed 86 dances from 70 bees, consisting of 24 different tremble dancers and 46 different waggle dancers. These bees danced a total of 61 waggle dances and 25 tremble dances and were respectively followed by 174 waggle followers and 64 tremble followers. Some of these videos were generated as part of research into honey bee stop signal communication (Nieh, 2010), but dancer and follower motions were never previously analyzed. All dances occurred on open worker cell combs.

### Measurements

Our goals were: (1) to determine when followers were first attracted to a dancer, (2) to quantify the subsequent behavior of followers and dancers, and (3) compare the behaviors of tremble and waggle followers. We videotaped returning foragers and analyzed their behavior and the behavior of their followers from the moment that following began. We determined the initial point of attraction, the time point at which a follower first began to follow. To do this, we played each video forward to confirm that the followers continued to pursue the dancer for at least 1 s. We then reversed the video, moving back frame-by-frame, to the time point of first attraction, defined as a worker turning towards or beginning to move closer to the dancer (methods of Tautz and Rohrseitz, 1998). We used custom software (available upon request from T.L.) to track both dancer and follower in 0.033 s intervals over 1 s (30 frames).

**Fig. 4.**
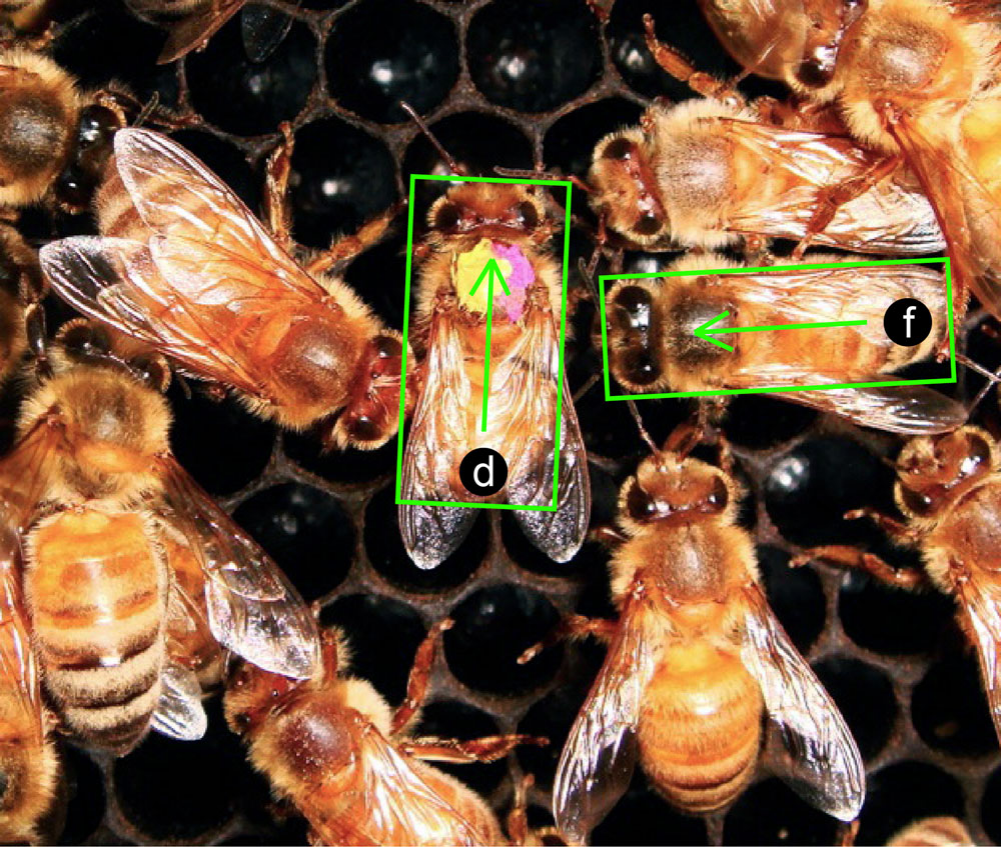
**Example frame from video tracking software showing how the dancer and a follower were marked.** Each arrow points towards the head, providing an orientation vector. d, dancer; f, follower; green box shows how bees were marked. The size of each box is scaled to match the body size (head-to-abdomen) of the subject.

In each frame, the dancer and a single follower were manually tracked by separately enclosing each bee in a scaled box delimited by the focal bee's head touching one edge (providing the forward vector direction) and the abdomen touching the opposite side (Fig. 4). The software generated a text file containing the frame number, the center positions of the dancer and the follower, the angle, and a size scaling factor for each box. We tracked only the dancer and one follower at a time, generating a separate file for each follower. For each waggle dance and each tremble dance, we tracked all followers whose initial point of attraction could be clearly identified. We tracked each waggle dance for an average of 2.7±2.5 s and each tremble dance for an average of 2.5±1.8 s.

These data were post-processed with Matlab R2013a (Mathworks). In some analyses, motion was calculated relative to the dancer, whose position was therefore fixed in each frame as the origin with a fixed angle of 0°. We defined the dancer-to-follower distance as the shortest distance between center tip of the follower's head and a line drawn along the midline of the dancer (Fig. 2A). To compensate for slight potential differences in video zoom, all trajectories were scaled to the same size, based upon the dancer body width. We measured change in relative position of the follower, preferences of follower position and orientation towards the dancer, linear and angular velocities of both dancer and follower.

To determine if follower behavioral differences were due to changes in bee densities or in camera zoom, we randomly selected one frame between the first and last frame of each video sequence and counted the number of visible bees and the number of cells (open worker cells in all videos) spanning the length and width of the video frame (4:3 fixed aspect ratio). On average, there were 1.0±0.3 bees cm^−2^ and the video frame covered an area of 166.5±76.4 cells^2^.

To measure the absolute motions of the dancers and followers, we focused on a subset of data (18 tremble dances from 18 different bees yielding 31 followers tracked for 0.6±0.3 s follower^−1^, and 44 waggle dances from 41 different bees yielding 96 followers tracked for 0.7±0.3 s follower^−1^) in which the camera did not move within 1 s. For each bee, we calculated forward velocity (movement of the bee forward along its longitudinal axis), sideways velocity (movement of the bee perpendicular to its longitudinal axis), and angular velocity (turning motions of the bee around its center). Forward velocity could be positive (movement forward) or negative (movement backward). Sideways velocity and angular velocity were analyzed as absolute values because no left versus right movement or turning biases were detected in our preliminary analyses.

The data showed potential differences in how followers oriented towards waggle dancers as compared to tremble dancers around the dancers' heads and abdomens. We therefore divided the space around a dancer's body into four quadrants. We enclosed the dancer in an ellipse with diameters of 14 mm and 5 mm. Our quadrants were chosen to divide this ellipse into four equal arcs of 15.88 mm in length, labeled as head, left, right, and abdomen (Fig. 3B). For every frame of each follower, we binned the position of the follower relative to the dancer into one of these four quadrants.

### Statistical analyses

We used repeated-measures analysis of covariance (ANCOVA), standard ANCOVA, or linear regression, as appropriate, to analyze our data, which met parametric assumptions as determined by residuals analysis. Repeated-measures were used to analyze data in which the same bee was tracked over time. We log transformed distance and angular velocity (deg s^−1^) data. We applied the arcsine square root transformation to the number of trophallaxis events per follower per dance. We included colony in our models as a random effect. We applied Tukey's honestly significant difference (HSD) tests for post hoc comparisons corrected for potential Type I statistical error.

To compare the mean densities of bees and the size of our video frames with the mean values reported by Tautz and Rohrseitz (1998), we used two-tailed Wilcoxon signed rank tests. For our quadrant analysis, we used nominal logistic regression (first approach data) and repeated-measures nominal logistic regression (approach data over time) and incorporated colony as a factor in both models.

We applied the Dunn-Sidak correction for Type I error in analyses when we tested the same data multiple times (*k*=number of multiple tests). Tests that remain significant after this correction are denoted ‘^DS^’. All statistical tests were performed with JMP v9 software and Microsoft Excel v14.6.1. We report mean±s.d. in the text. All measurement data are in Tables S1-S6.
